# Re-expansion of Thrombosed False Lumen after Aortic Dissection Due to Collateral Retrograde Flow from the Aortic Branches

**DOI:** 10.3400/avd.cr.21-00062

**Published:** 2021-12-25

**Authors:** Mamoru Hamuro, Senri Miwa, Kenji Yamamoto, Sakae Enomoto

**Affiliations:** 1 Department of Cardiovascular Surgery, Okamura Memorial Hospital, Sunto District, Shizuoka, Japan

**Keywords:** aortic dissection, tranexamic acid

## Abstract

Re-expansion of thrombosed false lumen after aortic dissection due to collateral retrograde flow from the aortic branches has rarely been reported. Surgical or endovascular local management such as ligation or occlusion of culprit arteries may not be effective in case retrograde blood flow to the false lumen might occur again from another branch after the operation. Here, we report a 68-year-old woman with re-expansion of the thrombosed false lumen after acute type B aortic dissection due to collateral retrograde flow from the aortic branches successfully treated with tranexamic acid therapy and antihypertensive therapy.

## Introduction

Uncomplicated acute type B aortic dissection is initially treated with medical therapy. Surgical management is considered for cases with life-threatening complications, including malperfusion syndrome, progression of dissection, enlarging aneurysm, or inability to control blood pressure or symptoms. Re-expansion of the thrombosed false lumen after aortic dissection due to collateral retrograde flow from the aortic branches has rarely been reported. The optimal treatment for this condition is unknown. We here report a 68-year-old woman with re-expansion of the thrombosed false lumen after acute type B aortic dissection caused by collateral retrograde flow from the aortic branches successfully treated with tranexamic acid therapy and antihypertensive therapy.

## Case Report

**Figure 1:**
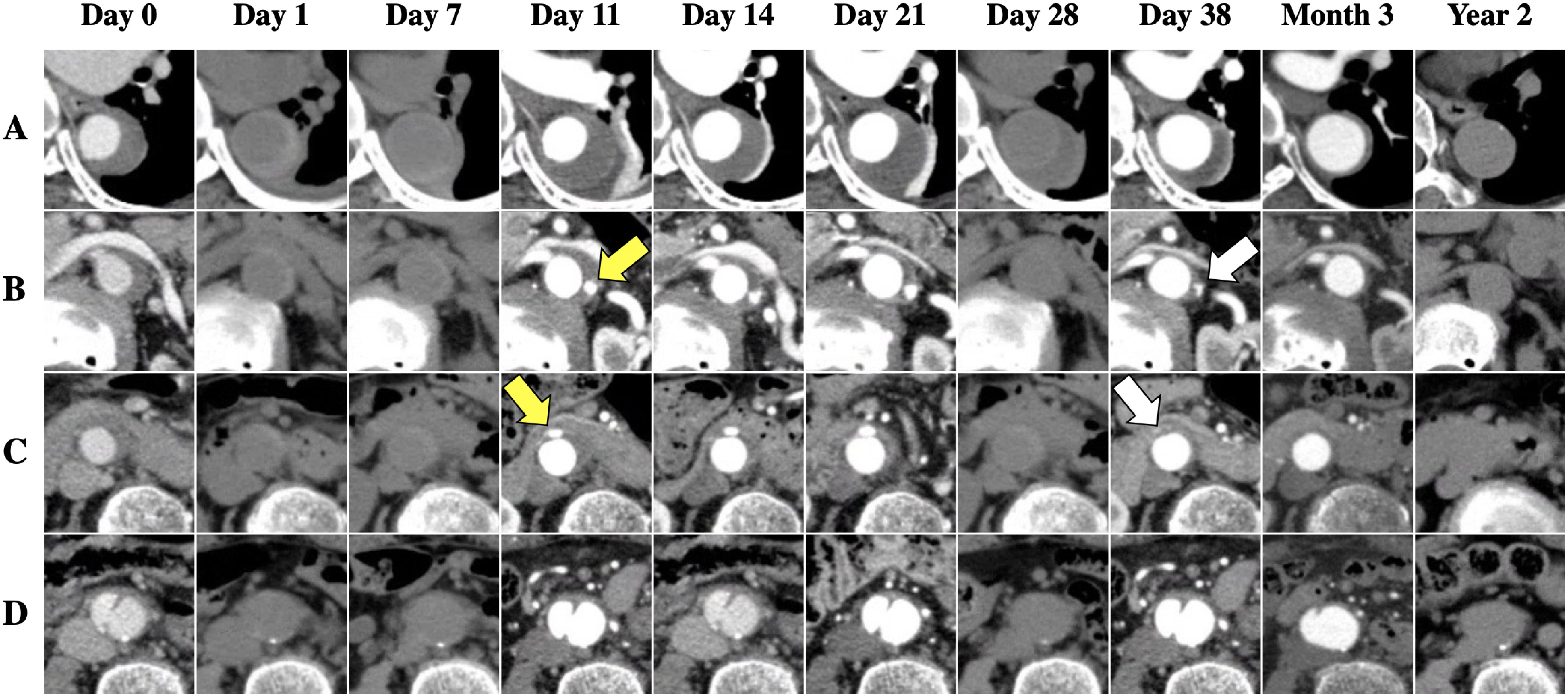
Fig. 1 Serial computed tomography (CT) scans (**A**: descending thoracic aorta level; **B**: left middle suprarenal artery level; **C**: right gonadal artery level; **D**: abdominal intimal defect level) show regression and re-expansion of the thrombosed false lumen and newly emerged collateral retrograde flow (yellow arrows) from the aortic branches on day 11. On day 38, enhanced CT demonstrate decreased flow from the left middle suprarenal artery, and that from the right gonadal artery disappeared (white arrows).

**Figure 2:**
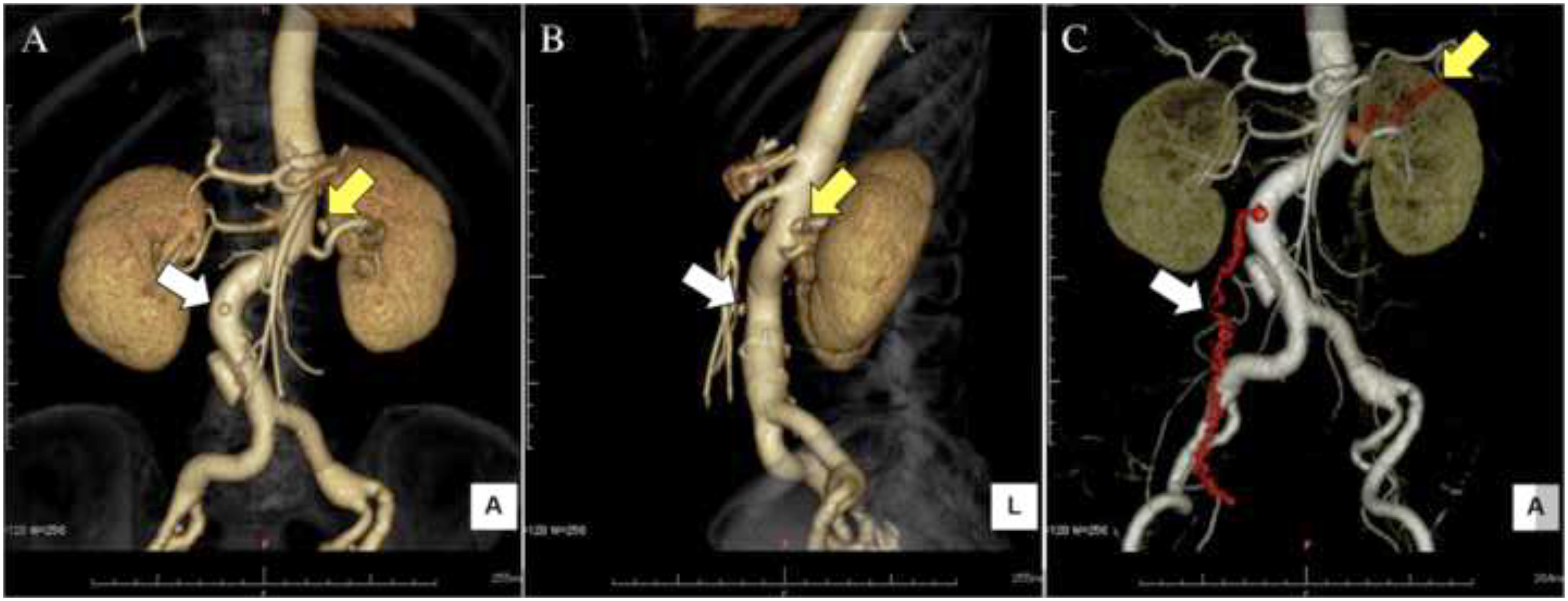
Fig. 2 Three-dimensional computed tomography (**A**, **C**: frontal view; **B**: lateral view) on day 11 show collateral retrograde flow from the aortic branches considered as the left middle suprarenal (yellow arrows) and right gonadal arteries (white arrows).

A 68-year-old woman complaining of dorsal pain was brought to our hospital. On admission, enhanced computed tomography (CT) revealed type B aortic dissection with predominantly thrombosed false lumen of the descending aorta. The penetrating atherosclerotic ulcer around an intimal tear of the abdominal aorta was assumed as the primary entry of the blood flow to the false lumen. There was no sign of complications of aortic dissection. Antihypertensive therapy, complete bed rest, pain relief, and the standard rehabilitation program were initiated according to the Japanese Circulation Society guidelines.^1)^ CT on the following day and after 7 days demonstrated regression of the false lumen. After initiating the rehabilitation program, the patients rest and activity levels were gradually increased. However, 11 days after admission, the patient complained of dorsal pain despite optimal antihypertensive therapy with less than 120 mmHg of systolic blood pressure. CT revealed re-expansion of the false lumen, and collateral retrograde flows to the thrombosed false lumen newly emerged from the aortic branches, which were considered the left middle suprarenal and right gonadal arteries. The false lumen around the abdominal intimal defect did not change (**Figs. 1** and **2**, and **Movie 1**). The patient was once again placed on bed rest and optimal antihypertensive therapy was continued; however, 14 days after admission, CT revealed false lumen re-expansion. Surgical or endovascular local management, such as ligation or occlusion, of culprit arteries was considered. However, in case retrograde blood flow to the false lumen occurs from other branches after the operation, another intervention might be needed again. Systemic medical treatment was selected. A second cycle of complete bed rest and oral tranexamic acid of 1,500 mg per day were initiated. After 21 days, CT revealed no false lumen re-expansion, and the resting level was gradually increased again. CT on postadmission day 28 revealed false lumen regression and transformation of the true lumen from ellipse to circle, indicating false lumen decompression. Furthermore, after 38 days, CT demonstrated decreased collateral retrograde flow to the thrombosed false lumen from the left middle suprarenal artery, and flow from the right gonadal artery disappeared. The false lumen was further regressed. Lastly, 40 days after admission, the patient was discharged with no complications. Three months later, CT revealed the disappearance of the thrombosed false lumen and CT 2 years later demonstrated no sign of recurrence.

## Discussion

Re-expansion of the thrombosed false lumen after aortic dissection due to collateral retrograde flow from the aortic branches has rarely been reported. Tranexamic acid therapy may be an effective treatment for this condition.

Aortic dissection is a life-threatening disorder, in which the aortic wall is detached into two layers at the medial level, causing two lumens having a certain length along the arterial course. Because uncomplicated Stanford type B acute aortic dissection follows a better natural course in acute phase than type A, initial management strategy is generally chosen as the medical treatment.^1,2)^ However, surgical treatment is necessary for patients with complications, such as malperfusion syndrome, progression of dissection, enlarging aneurysm, and inability to control blood pressure or symptoms because its prognosis is extremely poor.^3,4)^

Re-expansion of the thrombosed false lumen after aortic dissection caused by collateral retrograde flow from the aortic branches has rarely been reported. The optimal management for this condition is unknown. Wu et al. reported this condition as intramural blood pools in intramural hematomas.^5)^ They concluded that intramural blood pools were not associated with poor prognosis. In the report, however, 3 (27.3%) of 11 patients with new intramural blood pools at follow-up CT needed surgery. Intramural blood pools are not determined to be riskless conditions. In our case, the cause of the re-expansion could not be completely determined; however, retrograde blood flow was considered because the false lumen diminished in size just above the intimal tear and re-expansion was observed in more proximal zone. The middle suprarenal and gonadal arteries were collateral sources. Due to the re-expansion of the false lumen, local surgical or endovascular treatment to the culprit arteries such as ligation or embolization was considered. However, in case retrograde blood flow to the false lumen occurs from other branches after the operation, another intervention might be needed. Moreover, direct treatment may be difficult due to anatomical problems, depending on the type of branch. For these reasons, systemic medical treatment was selected.

Tranexamic acid therapy may be an effective treatment for thrombosed false lumen re-expansion due to collateral retrograde flow from aortic branches. Tranexamic acid is an antifibrinolytic agent that has been reported to be effective in treating enlarging aneurysms due to type II endoleak after endovascular repair.^6,7)^ In our case, tranexamic acid was used because the pathophysiological condition of retrograde blood flow from the branches to the thrombus in the enclosed space resembled a type II endoleak after endovascular aneurysm treatment. The cause of the occlusion of the branches was unknown. Optimal antihypertensive therapy was not effective, but after the initiation of tranexamic acid therapy, collateral retrograde flow to the thrombosed false lumen decreased, resulting in gradual regression of the false lumen. However, no similar report has been found. Thus, serial and thorough CT follow-up is important.

## Conclusion

Re-expansion of the thrombosed false lumen after aortic dissection due to collateral retrograde flow from aortic branches is rarely reported, and tranexamic acid therapy may be an effective treatment for this condition.

## Supplementary Information

Supplementary movie is available at the online article sites on J-STAGE and PMC.
